# Null Subcarrier Index Modulation in OFDM Systems for 6G and Beyond

**DOI:** 10.3390/s21217263

**Published:** 2021-10-31

**Authors:** Tuncay Eren, Aydin Akan

**Affiliations:** 1 Department of Electrical and Electronics Engineering, Istanbul University-Cerrahpasa, Istanbul 34320, Turkey; tun.eren@gmail.com; 2 Department of Electrical and Electronics Engineering, Izmir University of Economics, Izmir 35330, Turkey

**Keywords:** OFDM, index modulation, null subcarrier, computational complexity, 6G and beyond

## Abstract

Computational complexity is one of the drawbacks of orthogonal frequency division multiplexing (OFDM)-index modulation (IM) systems. In this study, a novel IM technique is proposed for OFDM systems by considering the null subcarrier locations (NSC-OFDM-IM) within a predetermined group in the frequency domain. So far, a variety of index modulation techniques have been proposed for OFDM systems. However, they are almost always based on modulating the active subcarrier indices. We propose a novel index modulation technique by employing the part of the transmitted bit group into the null subcarrier location index within the predefined size of the subgroup. The novelty comes from modulating null subcarriers rather than actives and reducing the computational complexity of the index selection and index detection algorithms at the transmitter and receiver, respectively. The proposed method is physically straightforward and easy to implement owing to the size of the subgroups, which is defined as a power of two. Based on the results of our simulations, it appeared that the proposed NSC-OFDM-IM does not suffer from any performance degradation compared to the existing OFDM-IM, while achieving better bit error rate (BER) performance and improved spectral efficiency (SE) compared to conventional OFDM. Moreover, in terms of computational complexity, the proposed approach has a significantly reduced complexity over the traditional OFDM-IM scheme.

## 1. Introduction

Rapid technological advancements in industry have resulted in an exponential increase in the number of connected devices, causing a massive volume of data traffic that appears almost impossible to manage by the current wireless networks. The OFDM waveform has been used in several ways and adopted well in several existing wireless communication systems. However, OFDM has certain disadvantages, such as high out-of-band radiation, high peak-to-average power ratio (PAPR) problems [1], and the loss of orthogonality caused by synchronization errors. Thus, the OFDM system requires some improvements to be used for future wireless systems. The primary aim of future-generation wireless communication systems is to provide three generic services: enhanced mobile broadband (eMBB) aiming to improve the high data rate, massive machine-type communications (mMTCs) concentrating on establishing connectivity among a large number of Internet of things (IoT) devices, and ultra-reliable low-latency communications (URLLCs) focusing on providing a link that is extremely sensitive and has a very low latency [2]. In order to meet the requirements of the aforementioned services, in addition to improving the performance of the OFDM system, several types of candidate waveforms for next-generation networks (6G and beyond) have been developed and studied in various environments such as industrial testbeds and academic research labs. In order to improve the performance of wireless systems in terms of spectral efficiency, energy efficiency, etc., the new candidate waveforms such as universal filtered multicarrier (UFMC) [3], generalized frequency division multiplexing (GFDM) [4], filter bank multicarrier (FBMC) [5], and filtered OFDM [6] have been studied. The key benefit of these candidate waveforms over the classical OFDM system is to improve the spectral efficiency losses caused by high out-of-band radiations.

On the other hand, new types of modulation techniques are proposed for OFDM systems to increase the data transmission ability of each subcarrier used in the frequency spectrum, while studies on the aforementioned new waveforms have also been in progress to meet the requirements of the services in 6G and beyond wireless networks. In addition to modulating the subcarriers by the complex constellation mapper, new research on the transmission of additional information bits that are conveyed by the indices of the active subcarriers have recently become very attractive. This new modulation form, known as index modulation (IM), was presented in [7] and has rapidly gained a broad interest. The main idea of IM is to transmit the data not only by modulating the subcarriers, but also by the carrier indices [8], which are involved in transmission based on the incoming data stream.

Although, in some configurations, OFDM-IM improves the spectral efficiency over the OFDM systems, the computational complexity of the OFDM-IM waveform remains an open issue. To tackle this issue, several studies have been carried out to reduce the complexity of the system at the transmitter and receiver. An energy-based greedy detection method was proposed with reduced complexity maximum likelihood (ML) in [9]. A modified frequency index modulation (FIM) scheme, which aims to reduce the transmitted energy, was suggested in [10]. This system has a lower complexity compared to the OFDM-IM scheme. By specifying a threshold for the likelihood probability, Reference [11] provided a simpler “log-likelihood ratio (LLR)” calculation approach for OFDM-IM that delivers near-optimal coded BER performance, with significantly less complexity. As for the low-complexity receiver design, two linear complexity detection schemes taking into account the minimum mean-squared error and zero-forcing receivers were proposed in [12]. A generalized time domain index modulation scheme (GTD-IM) with low complexity was introduced in [13], where the receiver uses a square-law envelope detector (SLED) to detect the active indices. Furthermore, recently, the idea of using deep learning to detect data bits in the OFDM-IM system was proposed [14]. The suggested detector has approximately the same performance as the ML detection, but significantly reduces the runtime of existing detectors in classical OFDM-IM. The detector in [15], inspired by the sphere decoding approach, was designed for subblock-design-aided OFDM with all-index modulation (SD-OFDM-AIM). In [16], by using the same set of active subcarrier indices in two distinguishable clusters and implementing coordinate interleaving for M-ary modulated symbols, the repeated index modulation OFDM with coordinate interleaving (RIM-OFDM-CI) was presented. In [17], a low-complexity hybrid number and index modulation (OFDM-HNIM) scheme was developed for transmitting additional information bits using the number and index of active subcarriers. However, all the above studies were proposed for conveying the bits to the indices of active subcarriers in complex subgroups, and they employed either look-up tables or combinatorial methods for index mapping and detection algorithms.

In this study, a new index modulation scheme for OFDM systems is introduced. The modulation is performed at the positions of the nulled subcarriers in the frequency domain. Contrary to the existing OFDM-IM techniques, where the index modulation takes place on the active subcarriers, in this new modulation method, the information bits are conveyed by the indices of the nulled subcarriers. This new method significantly reduces the number of computations as compared to the existing OFDM-IM schemes. It is worth noting that, to the best of our knowledge, this is the first study where the index modulation process was carried out by utilizing null subcarriers without the need for using look-up tables or combinatoric algorithms for the index selection and detection at the transmitter and receiver, respectively.

### Contributions and Organization

The main goal of this research was to design a new scheme for reducing the computational complexity of the OFDM-IM systems by utilizing null subcarrier locations rather than active subcarriers. The major contributions of this paper are summarized as follows:

- The proposed NSC-OFDM-IM scheme algorithm starts by assuming all the subcarriers as if they are active in the subgroup. Depending on the incoming bit substream, one subcarrier among them is set to inactive. On the other hand, conventional OFDM-IM first assumes all subcarriers as if they are inactive in the subgroup, and the index selector algorithm starts by targeting the combination (or sequences) of activated subcarriers based on lexicographic order in the complex subgroup;
- *Transmitter complexity and index selection:* In conventional OFDM-IM, in order to determine the indices of the active subcarriers, the first step is to convert the index bits into their equivalent natural value, and then, the combination of k indices corresponding to this natural number is obtained using the combinatorial algorithm. The combinatorial (or combinadics) algorithm is used in existing methods to perform a one-to-one mapping between the decimal number of the index bits and k combinations. In other words, the OFDM-IM scheme requires a number of combinatorial processing in order for the index selector to be able to find the corresponding indices of the subcarriers within the complex subgroup. However, in our proposed method, the index bits are first converted to their corresponding decimal value, and then, the index selector in this scheme calculates the one greater than this decimal value. The new value is then set as the inactive carrier position in the frequency subgroup. In other words, the NSC-OFDM-IM index selector just functions by deactivating one of the active subcarriers in the complex subgroup based on the decimal value of the incoming index bits. By comparing the computational complexity of the index selection mechanisms of the conventional and the proposed schemes, it was found that in the proposed NSC-OFDM-IM, the index selection is performed very easily just by adding 1 to the corresponding decimal value of the index bits, and hence, the proposed method has a significantly reduced complexity at the transmitter;
- *Receiver complexity and index detection:* At the receiver, after the signal is converted to frequency domain representation, the LLR processing is applied to both conventional OFDM-IM and the proposed NSC-OFDM-IM in the same way. However, the complexity reduction of the index detection algorithm in the proposed scheme comes from the following two aspects:
  1. The first complexity reduction is eliminating the need to apply a sorting algorithm to the coefficients in the LLR subgroups. Hence, there is no sorting complexity in all LLR subgroups;
  2. In comparison to the conventional scheme, the use of the combinadic ranking algorithm as part of the index detection process is no longer essential in the proposed method because no sorting in the LLR subgroups is required. Hence, there is no complexity in terms of finding the indices of the active carrier locations.
  The detection mechanism in the proposed method just finds the index of the minimum value of the coefficients in each LLR subgroup. The receiver detector then takes one less than the index number and uses it as the decimal number to convert to the binary value.

The remainder of the paper is structured as follows: The conventional OFDM system is summarized in Section 2. The existing OFDM-IM technique is discussed in Section 3. The proposed novel NSC-IM for OFDM system (NSC-OFDM-IM) is introduced in Section 4. The multipath channel model as a transmission environment utilized in this study is given in Section 5. The complexity of the proposed NSC-OFDM-IM scheme is compared with the OFDM-IM scheme in Section 6. The computer simulation results are evaluated in Section 7. Following the discussions on the experimental results in Section 8, the paper is ended by the Conclusions in Section 9.

## 2. Conventional OFDM Systems

Orthogonal frequency division multiplexing (OFDM) is a multicarrier transmission technique that is widely used in wireless communication systems. In the Long-Term Evolution (LTE) systems, OFDM is the key waveform constructed in the in-parallel form of orthogonal subcarriers. The subcarriers’ orthogonality is achieved by carefully choosing each carrier frequency in the spectrum. As a basic concept, the available frequency bandwidth is divided into a number of narrowband frequencies, and they are positioned orthogonal to one another along the spectrum [18]. In contrast to the traditional frequency division multiplexing (FDM) schemes, in the OFDM system, the subcarriers that use the allocated frequency bandwidth are allowed to overlap each other, and thus, more carriers are deployed in the transmission.

**Figure 1:**
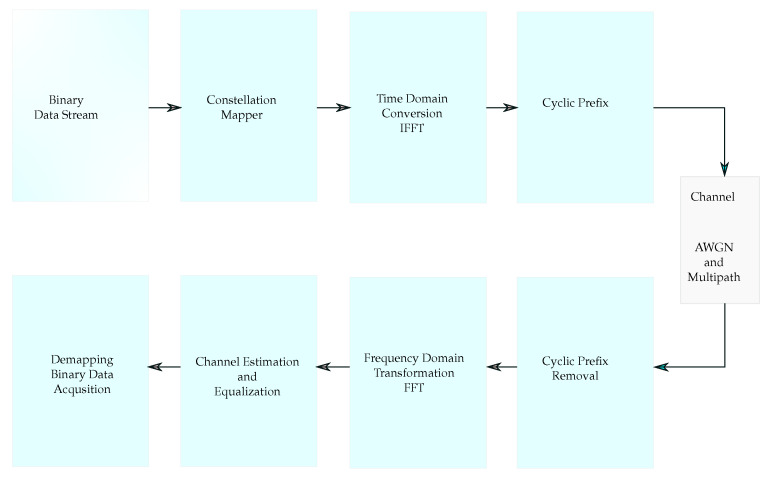
Conventional OFDM system.

The basic diagram of the OFDM system is shown in Figure 1. It consists of the transmitter, receiver, and wireless communication channel.

The binary data are given to the constellation mapper, and then, they are converted to the complex data. The constellation mapper operates with a predefined modulation order as a power of two (2, 4, 8, etc.). The complex data at the output of the constellation mapper are used for the frequency domain representation. The inverse fast Fourier transform (IFFT) block is fed by the constellation outputs, and by processing the complex inputs, the orthogonal subcarriers in the time domain are obtained. Each subcarrier at a different center frequency is modulated by the IFFT to carry the complex data. The OFDM symbol is constructed by adding the Cyclic Prefix (CP), also known as the guard time interval, at the beginning of the signal. The CP insertion allows for more efficient communication against the intersymbol interference (ISI).

The OFDM signal generated in the time domain can be expressed as:

(1) $$\begin{array}{cc} x(\ell ) & =IDFT\{X(k)\}\;\ell =0,1,\cdots ,N-1\\ \; & =\frac{1}{\sqrt{N}}\sum_{k=0}^{N-1}X\left(k\right)e^{j\frac{2\pi k}{N}\ell } \end{array}$$

where IDFT indicates the inverse discrete Fourier transform, $\frac{1}{\sqrt{N}}$ denotes the scaling factor, and the time domain OFDM symbol samples are indicated by x(ℓ), where ℓ=0,⋯,N−1, for each subcarrier. The subcarrier index k=0,⋯,N−1, represents the frequency domain samples.

The OFDM guard interval can be inserted in a number of different ways: one of them is the time domain zero padding (ZP), which pads the signal with zero samples, while the other is the cyclic prefix addition, where the last $N_{CP}$ samples of the signal are copied and then inserted into the front of the OFDM time signal after the IFFT process, which modulates the subcarriers with the complex data [19].

By the CP addition, the OFDM signal x(ℓ) is then extended to the length of $N+N_{cp}$ before the transmission. The extended version of the OFDM signal $x_{cp}\left(\ell \right)$ is given by:

(2) $$x_{cp}\left(\ell \right)=\left\{\begin{array}{cc} x(N+\ell ), & \ell =-N_{cp},-N_{cp}+1,\cdots ,-1\\ x(\ell ), & \ell =0,1,2,\cdots ,N-1 \end{array}\right.$$

The advantage of adding a guard interval to the OFDM symbols is that, with a sufficiently long cyclic prefix, the periodicity of subcarriers remains alive, and thus, it allows more efficient communication against ISI [20]. In a multipath channel environment, the length of the OFDM cyclic prefix is chosen to be equal to or greater than the length of the maximum path delay [21].

At the receiver, the OFDM signal y(ℓ) is received from the wireless channel, and then, the frequency domain representation of the signal is obtained, following the guard interval removal.

The signal y(ℓ) in Equation (3) is the convolution of the transmitted OFDM signal x(ℓ) and the channel impulse response h(ℓ).

(3) $$\begin{array}{cc} y(\ell ) & =x(\ell )*h(\ell )+w(\ell )\\ Y(k) & =X(k)H(k)+W(k) \end{array}$$

where w(ℓ) denotes additive white Gaussian noise (AWGN) and W(k) is the frequency domain representation of the noise vector w(ℓ). The frequency domain representation Y(k) of the received signal is given by,

(4) $$\begin{array}{cc} Y(k) & =DFT\{y(\ell )\}\;k=0,1,\cdots ,N-1\\ \; & =\sqrt{N}\sum_{k=0}^{N-1}y\left(\ell \right)e^{-j\frac{2\pi k}{N}\ell } \end{array}$$

where DFT indicates the discrete Fourier transform operation.

## 3. Index Modulation for OFDM Systems

**Figure 2:**
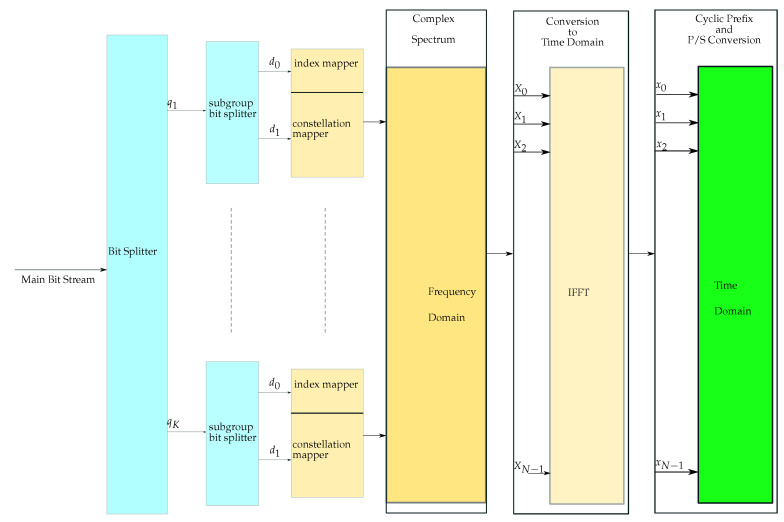
OFDM-IM system transmitter.

The transmission of information through indices was originally carried out by using the spatial modulation (SM) technique [22,23]. The SM method was then utilized in OFDM systems by allowing it to transmit additional data bits by modulating the indices of the active subcarriers [24]. However, in this method, the number of active subcarriers changes between the consecutive OFDM symbols. Thus, in order to make a suitable design of the active subcarriers and enhance the performance of the previous approaches, a new technique called OFDM-IM was introduced in [7]. The primary concept of OFDM-IM is to use the active subcarrier indices for additional data transmission besides modulating the same active subcarriers with complex data [25,26]. The basic OFDM-IM transmitter diagram is given in Figure 2.

The incoming bit stream is divided into a number of *K* groups, each containing a predetermined number of *q* bits according to the complex modulation order used in the system and the number of used *k* active subcarriers in the frequency domain complex subgroups. The total number of complex subgroups in the frequency domain is also equal to *K* such that K=N/n, where *N* is the number of total subcarrier locations in the frequency axis, regardless of whether they are active or inactive for the complex and index modulations, and *n* denotes the size of the complex subgroups.

Then, each group of bits is divided into two separate subgroups within itself, containing $d_{0}$ and $d_{1}$ bits. In other words, one subgroup with $d_{0}$ bits is used to modulate the indices of the active subcarriers, while the other $d_{1}$ bits are converted into complex data via the constellation mapper and used to modulate the active subcarriers as in the classical OFDM method.

The number of $d_{0}$ bits is determined by the size of the complex subgroup and the number of active subcarriers within the complex subgroup. In other words, the number of bits that come with the $d_{0}$ bit sequence is determined by the combinational selection number (C(n,k)), where all possible selections of *k* active subcarriers in the subgroup are obtained [27,28].

The $d_{0}$ bits, which are used for selecting and modulating the indices of the active subcarriers, can be represented as:

(5) $$d_{0}=\left\lfloor log_{2}\left(C\left(n,k\right)\right)\right\rfloor$$

The $d_{1}$ bits for the number of *k* activated subcarriers are determined according to the order of the constellation mapper. The number of $d_{1}$ bits is obtained as follows:

(6) $$d_{1}=klog_{2}\left(M\right)$$

so the number of *q* bits per subgroup, which are conveyed via the indices of the active subcarriers and mapped to the complex data for the modulation of the phases of the active subcarriers, can be represented as:

(7) $$q=d_{0}+d_{1}$$

then the total number of bits transmitted over one symbol is given by:

(8) $$\begin{array}{cc} m & =K(d_{0}+d_{1})\\ \; & =\frac{N}{n}\left(\left(d_{0}+d_{1}\right)\right) \end{array}$$

Each complex subgroup is designed to have the number of *n* subcarrier locations; *q* is the number of total bits to be transmitted by the subcarriers in one complex subgroup.

The index selector at the transmitter uses an unranking combination algorithm [29] to be able to produce the corresponding *k*-size subset vector from the nk combinations. By using the vector elements, the index selector specifies the number of active subcarriers in the complex subgroup, where *k* of *n* subcarriers are activated [30]. Hence, the active subcarriers are the source of the information in the subgroup and transmit the data via their indices and phases [31].

At the receiver, the signal is analyzed in the frequency domain to make the detection of the transmitted bits. The data are extracted from the indices and phases of the active subcarriers. In order to obtain the indices of active subcarriers, the maximum likelihood (ML) or LLR detection methods are employed for the OFDM-IM system [32,33]. The receiver then uses the combinadic ranking algorithm to extract the corresponding decimal number of the data conveyed by the indices [29].

## 4. Proposed NSC-OFDM-IM

**Figure 3:**
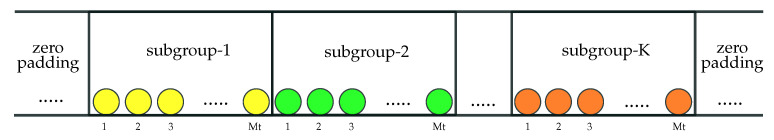
The subgroups in the frequency domain.

The OFDM symbol in the frequency domain is grouped into a number of sub-bands in Figure 3, while each has the same number of subcarrier indices. The size of the frequency sub-bands is determined as a power of two, which can also be equal to the order of the constellation mapper in the system. In the proposed scheme, one carrier is set to the inactive state within each sub-band group based on the incoming bit stream, and the others are used as the active subcarriers, which are modulated by the complex data after the conversion of the binary data at the output of the constellation mapper.

**Figure 4:**
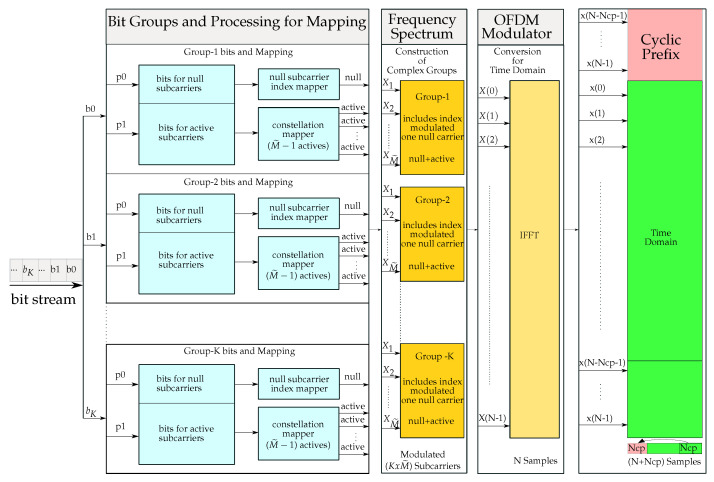
The proposed NSC-OFDM-IM transmitter.

The transmitter of the proposed NSC-OFDM-IM scheme is illustrated in Figure 4. The bit stream is first split into $b_{\kappa }$ main groups, and then, each $b_{\kappa }$ bit group is further divided into two subgroups with a different number of bits. The first subgroup contains the number of $p_{0}$ bits as per the order of the index mapper, which is predefined as a power of two based on the size of the frequency domain sub-band groups. These $p_{0}$ bits are used to modulate the zero subcarrier index within the OFDM frequency sub-band. The second bit subgroup is designed to have $p_{1}$ bits to be used for the modulation of the active subcarriers in the complex subgroup, as in a conventional OFDM system.

The number of $p_{0}$ and $p_{1}$ bits, which are split from the $b_{\kappa }$ main group, are then given by,

(9) $$\begin{array}{cc} p_{0} & =log_{2}\tilde{M}\\ p_{1} & =\left(\tilde{M}-1\right)log_{2}M \end{array}$$

where $\tilde{M}$ denotes the size of complex sub-band group as a power of two, which is $2^{p_{0}}$ in this case, and *M* is the modulation order by means of the constellation mapper.

$\tilde{M}$ can also be called the null subcarrier index mapper order (IMO) since the number of $p_{0}$ bits is conveyed to the inactive carrier index number. The number of bits in one of $b_{\kappa }$ main bit groups is then defined as,

(10) $$\begin{array}{cc} b_{1} & =p_{0}+p_{1}\\ \; & =log_{2}\tilde{M}+\left(\tilde{M}-1\right)log_{2}M \end{array}$$

where $b_{1}$ indicates the first bit group of the main bit stream.

The transmitted bits in one NSC-OFDM-IM symbol consist of all $b_{\kappa }$ bit groups such that:

(11) $$b_{\kappa }=\left\{b_{1},b_{2},\cdots ,b_{K}\right\}$$

Then, the total number of *m* bits carried by the null subcarrier indices and active subcarrier phases in the NSC-OFDM-IM is given by:

(12) $$m=K(p_{0}+p_{1})$$

where *K* denotes the total number of $b_{\kappa }$ main bit groups, which is also equal to the number of frequency domain complex sub-band groups.

The subcarrier indices in the complex group are given by,

(13) $$X=\{X_{1},X_{2},\cdots ,X_{\tilde{M}}\}$$

The index of the null subcarrier within the complex sub-band group is easily selected by the index mapper, which converts the binary $p_{0}$ data to the decimal value. Based on the decimal value of the $p_{0}$ bit sequence, the index mapper then finds the corresponding carrier location that is equal to one more than the decimal value of $p_{0}$ bits as defined in Table 1. Upon finding the frequency location in the complex subgroup, the subcarrier in this group is set to an inactive state. In other words, there is no active carrier generated at this frequency, and hence, it will be a null subcarrier in the NSC-OFDM-IM symbol. The remaining subcarriers will be set as the active carriers and modulated by the complex dataset, which is generated by the constellation mapper, as in the classical OFDM systems.

The corresponding NSC-OFDM-IM symbol in the frequency domain is then constructed by the complex subgroups. The frequency domain representation of the available bandwidth for the proposed scheme is given by:

(14) X={X(0),X(1),X(2),⋯,X(N−1)}

where *N* denotes the size of the IFFT/FFT, which can also be called $N_{FFT}$, in the system.

In the case of employing all carrier positions, regardless of whether they are active or inactive subcarriers, they all carry the information by index and complex modulations. The number of *K* complex subgroups is given by:

(15) $$K=\frac{N_{FFT}}{\tilde{M}}$$

If the number of used frequency locations $N_{used}$ is less than the OFDM spectrum, this means the system is padded with zeros in the frequency domain, and as in the classical zero-padded OFDM (ZP-OFDM) symbol, the proposed scheme here can be interpreted as the null subcarrier index modulation for the zero-padded OFDM system (NSC-ZP-OFDM-IM).

Depending on the decimal value of the $p_{0}$ bits, the selection of the inactive carrier index within the complex sub-band group occurs randomly, and hence, the active carriers in each sub-band in the frequency domain can be interpreted as sparsely distributed active subcarriers.

The remaining part of the NSC-OFDM-IM scheme at the transmitter is designed as a classical OFDM system. The complex modulation and guard interval addition are applied accordingly. The signal is then given to the wireless multipath channel.

### 4.1. Receiver Design and Detection

**Figure 5:**
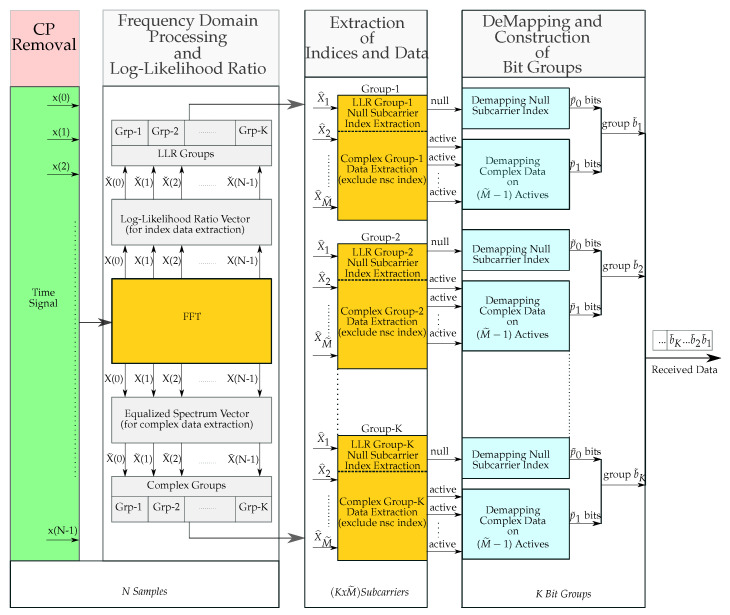
The proposed NSC-OFDM-IM receiver.

The NSC-OFDM-IM receiver system was designed as illustrated in Figure 5. The guard interval is removed from the time domain received signal, and then, it is fed into the FFT block for frequency domain representation. Since the transmit signal is affected by the wireless channel environment, it is necessary to remove or, at least, to minimize the channel disturbance from the received signal.

The frequency domain representation of the signal contains two types of data: one is the complex data embedded in the phases of the active subcarriers, and the other one is the index data conveyed by the indices of the null subcarriers. Thus, we first need to obtain the indices of the inactive subcarriers along the OFDM spectrum, and then, the complex data locations over active subcarriers are extracted by excluding the null carrier locations. To do so, in our framework, after applying the FFT process to the time domain received signal, the complex signal vector is processed in two parallel ways: one is to construct the log-likelihood ratio vector to be used for obtaining the null carrier indices, and the other is to equalize the frequency domain signal by using the channel frequency response vector to achieve the complex modulated data.

The frequency domain equalization is an easy task to remove the channel effect since it functions over each carrier separately. In case the channel is unknown at the receiver side, before the equalization, one should take into consideration that the wireless channel impulse response should be estimated to properly equalize the frequency domain received signal. Let us consider that the receiver has the perfect channel knowledge and thus does not need a channel estimation process. In this case, the signal is equalized by a one-tap equalizer, and now, the received signal in the N-point frequency domain is ready for constructing the complex domain subgroups.

After applying the frequency domain equalization, the complex data are divided into subgroups with a size of $\tilde{M}$. The indices of the equalized subcarriers in the complex groups are given by,

(16) $$\hat{X}=\left\{{\hat{X}}_{1},{\hat{X}}_{2},\cdots ,{\hat{X}}_{\tilde{M}}\right\}$$

#### 4.1.1. Log-Likelihood Ratio Processing

The next step is to find out the index of the inactive carrier location within each complex subgroup. As we explained at the transmitter side, the inactive carrier location is left as zero, and no carrier frequency is generated at this frequency location. Hence, at the receiver side, this allows us to design a very flexible and easily applicable detection mechanism with lower computational complexity.

In order to obtain the null carrier indices, the frequency domain signal is again processed separately, and thus, the log-likelihood ratio detection mechanism is applied to the complex signal vector.

The LLR detector in the proposed NSC-OFDM-IM scheme provides the logarithm of the ratio of the a posteriori probabilities of the frequency domain signal vector, and it works by considering all frequency locations, regardless of whether they are active or inactive, along the frequency axis. The ratio of these a posteriori probabilities is given by,

(17) $$\lambda \left(\beta \right)=ln\frac{\sum_{\kappa =0}^{M}P\left(X\left(\beta \right)=s_{\kappa }\right|y_{F}\left(\beta \right)}{P(X\left(\beta \right)=0|y_{F}\left(\beta \right)}\;\beta =1,2,\cdots ,N$$

As a simplified representation of this ratio, by using the Bayesian theorem, the λ(β) can be expressed for BPSK-modulated NSC-OFDM-IM as below:

(18) $$\lambda \left(\beta \right)=max\left(a,b\right)+ln(1+exp(-|b-a|))+\frac{|y_{F}{\left(\beta \right)|}^{2}}{N_{0,F}}$$

where $a=-|y_{F}\left(\beta \right)-h_{F}{\left(\beta \right)|}^{2}/N_{0,F}$ and $b=-|y_{F}\left(\beta \right)+h_{F}{\left(\beta \right)|}^{2}/N_{0,F}$. $N_{0,F}$ denotes the noise variance in the frequency domain, and it can be expressed as,

(19) $$N_{0,F}=\left(\frac{N_{used}}{N_{FFT}}\right)N_{0,T}$$

where $N_{used}$ denotes the number of used active subcarriers in the frequency spectrum.

After applying the log-likelihood ratio to the frequency signal vector at the output of FFT processing, the LLR vector with a length of N is achieved, and now, it is ready for subgroup separation. The size of the LLR subgroups is the same as the complex subgroups. The main objective of constructing these subgroups in the LLR domain is to find out the null carrier index within each subgroup so that the index modulated data are extracted from the received signal.

The coefficients in the LLR vector are given by,

(20) $$\overset{ˇ}{X}=\left\{\overset{ˇ}{X}\left(1\right),\overset{ˇ}{X}\left(2\right),\cdots ,\overset{ˇ}{X}\left(N\right)\right\}$$

At this stage, we have two types of subgroups with the same size $\tilde{M}$, which are complex subgroups for complex data extraction, and LLR subgroups to be used for index data extraction. These subgroups are processed separately in parallel form.

For example, the first null carrier index is obtained from the first LLR subgroup, and in the meantime, by excluding this null carrier index, the active subcarriers are extracted from the first complex subgroup.

As we designed at the transmitter side, each complex subgroup contains one null subcarrier, which is used for conveying the index data, and $\left(\tilde{M}-1\right)$ active subcarriers, which are used for complex data transmission. With this in mind, after the LLR calculation and subgroup separation in the LLR domain, the values in each LLR subgroup can be interpreted as the likelihood ratio, with a low value, which indicates that the observed result is unlikely to occur, which means that the occurrence of the null subcarrier at this location is highly possible, and with the remaining values, which can be interpreted as the occurrence of the active subcarriers.

The interpretation of LLR values in each subgroup allows us to easily extract the null carrier location among the $\tilde{M}$ carrier locations. To do so, each subgroup is evaluated separately in terms of its minimum real value, which indicates the null carrier position in the complex subgroup. As an example, let us define the index of the minimum real value in the first LLR subgroup, which is given by,

(21) $$\overset{ˇ}{X}\left(z\right)=\left\{\overset{ˇ}{X}\left(1\right),\overset{ˇ}{X}\left(2\right),\ldots ,\overset{ˇ}{X}\left(\tilde{M}\right)\right\}$$

Then, the index, which indicates the minimum LLR value in the first subgroup, can be expressed as,

(22) $$\tilde{i}=\arg\min_{z}\overset{ˇ}{X}\left(z\right)$$

The proposed NSC-OFDM-IM index demapper is given in Table 2.

The index of the minimum LLR value corresponds to the null carrier location in the subgroup, and by using the index $\tilde{i}$, we easily calculate the decimal value of the corresponding bit sequence. The decimal value of the transmitted bit sequence is expressed as,

(23) $$\tilde{z}=\tilde{i}-1$$

The decimal value $\tilde{z}$ is converted to binary, resulting in the bit ${\tilde{p}}_{0}$ sequence. In order to obtain the complex data from the active subcarriers, we first exclude the null carrier index from the frequency domain subgroup, and then, based on the order of the complex constellation mapper, the complex demapping process takes place to obtain the transmitted bit sequence ${\tilde{p}}_{1}$.

The number of bits, ${\tilde{b}}_{\kappa }$, per each complex group, and the total number of received bits, $\tilde{m}$, are defined as,

(24) $$\begin{array}{cc} {\tilde{b}}_{\kappa } & ={\tilde{p}}_{0}+{\tilde{p}}_{1}\\ \tilde{m} & =\{{\tilde{b}}_{1},{\tilde{b}}_{2},\cdots ,{\tilde{b}}_{K}\}\\ \; & =K({\tilde{p}}_{0}+{\tilde{p}}_{1}) \end{array}$$

#### 4.1.2. Visualization of the Proposed NSC-OFDM-IM Scheme

**Figure 6:**
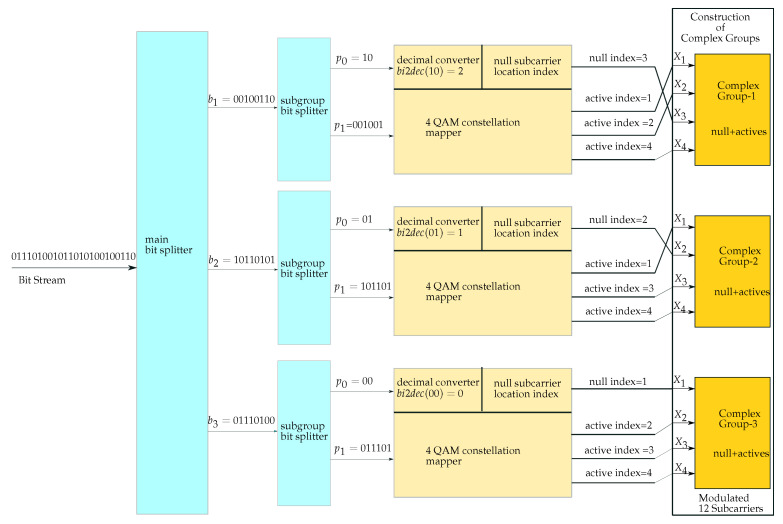
Example of the NSC-OFDM-IM transmitter.

For the sake of the clear understanding and visualization of the proposed NSC-OFDM-IM scheme, the following illustrative example is given in Figure 6.

**Figure 7:**
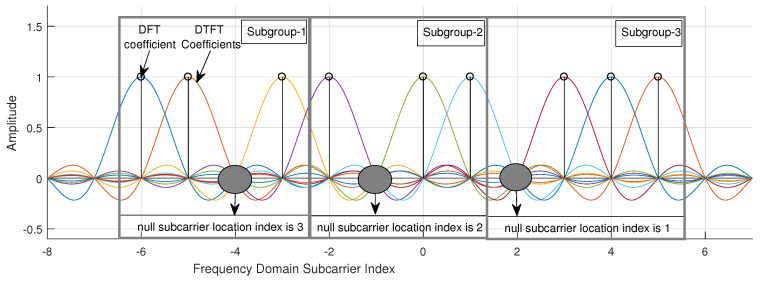
Frequency domain illustration of the modulated example carriers.

Let us suppose that we have a bit stream that will be sent over 12 subcarrier frequency points, regardless of whether they are set to an active or inactive state. The frequency domain localization of the active and inactive subcarriers for the given example is illustrated in Figure 7.

The bit stream is divided into three subgroups. This means that we have three complex subgroups, and each has four consecutive carrier locations in the frequency domain. In each bit subgroup, the first $p_{0}$ bits are used for the null carrier index, and the remaining $p_{1}$ bits are used for complex mapping. The $p_{0}$ bits used in the given example are {10}, which are in the first subgroup, {01}, which are in the second subgroup, and {00}, which are in the third subgroup. They are first converted to their corresponding decimal value and then fed into the index mapper to obtain the indices of the null subcarrier locations in the complex subgroups.

After the decimal conversion of the $p_{0}$ bits, the corresponding indices of the null subcarriers are obtained as 3 in the first complex subgroup, 2 in the second complex subgroup, and 1 in the third complex group. The remaining subcarrier indices in the subgroups are {1,2,4} in the first complex group, {1,3,4} in the second complex group, and {2,3,4} in the third complex group. They indicate the locations of the active subcarriers in the frequency domain. In the given example, the corresponding complex data of the $p_{1}$ bits modulate these active subcarriers.

### 4.2. Spectral Efficiency

The spectral efficiency (SE) is an important measurement parameter in OFDM systems, and it is defined as the symbol transmission rate in a unit band [34,35]. For a conventional OFDM system, the SE can be expressed as,

(25) $$SE_{OFDM}=\frac{N_{used}\left(log_{2}M\right)}{N_{FFT}+N_{cp}}$$

where *M* denotes the modulation order of the constellation mapper, $N_{used}$ is the number of subcarriers used for data transmission, and $N_{cp}$ is the length of the cyclic prefix.

For the existing OFDM-IM and our proposed NSC-OFDM-IM schemes, the SE can be represented as,

(26) $$SE_{OFDM-IM}=\frac{K(k\left(log_{2}M)+\left\lfloor log_{2}\left(C\left(n,k\right)\right)\right\rfloor \right)}{N_{FFT}+N_{cp}}$$

(27) $$SE_{NSC-OFDM-IM}=\frac{K(log_{2}\tilde{M}+\left(\tilde{M}-1\right)log_{2}M)}{N_{FFT}+N_{cp}}$$

where *n* and *k* denote the size of the complex subgroup and the number of active carriers in each subgroup for the OFDM-IM scheme, respectively. $\tilde{M}$ is the index modulation order (or subgroup size) for the NSC-OFDM-IM scheme. *K* is the number of complex subgroups in the frequency domain.

The performance analysis in terms of the spectral efficiency of the proposed scheme is compared with the conventional OFDM and OFDM-IM schemes in Table 3.

As can be seen from Table 3, based on the size of the complex subgroups and the constellation mapper order of the system, the proposed NSC-OFDM-IM scheme exhibits an improved SE over the conventional OFDM scheme while achieving the same SE as compared to the OFDM-IM scheme. The maximum spectral efficiencies (the green area in Table 3) are obtained for BSK modulation by using the subgroups with 4 and 8 subcarriers, for 4-QAM modulation by using the subgroups with 8 and 16 subcarriers, and for 8-PSK modulation by using the subgroups with 16 and 32 subcarriers. The proposed NSC-OFDM-IM scheme exhibits the same SE (the gray area in Table 3) as the conventional OFDM systems when the index modulation order $\tilde{M}$ equals the complex modulation order *M*.

The succession rate of obtaining the correct indices of the null subcarriers in the complex subgroups is given by:

(28) $$\begin{array}{cc} \eta & =\frac{1}{N_{sym}}\sum_{\alpha =1}^{N_{sym}}\frac{K-\epsilon_{\alpha }}{K} \end{array}$$

where η denotes the succession rate of the null carrier indices per the SNR and $\epsilon_{\alpha }$ is the number of failed indices.

In the increasing SNR values, the null subcarriers in the subgroups are successfully captured, and the value of the succession rate η converges to one.

## 5. Multipath Channel Model

The signal propagating through the wireless channel environment experiences physical changes such as dispersion, refraction, and scattering. Hence, the received signal disturbed by the wireless channel objects has the multiple copies of the originally transmitted signal with different phases and amplitudes. The impulse response of the multipath Rayleigh fading channel through which the OFDM signal passes can be characterized as follows:

(29) $$h\left(\tau ,t\right)=\sum_{l=0}^{L-1}\alpha_{l}\left(t\right)\delta (t-\tau_{l}\left(t\right))$$

where *L* denotes the number of taps, τ is the delay time of each direction, and α is the complex coefficient of each path.

## 6. Complexity Analysis

Computational complexity is one of the most critical challenges in the design of the transmitter and receiver algorithms in wireless communication systems:

- Transmitter complexity: As mentioned previously, in the OFDM-IM scheme, the indices of the active subcarriers are determined based on the lexicographical order of the corresponding decimal value of the $p_{0}$ index bits. In order for the index selector to obtain the k-size vector of indexes out of $2^{length(p_{0})}$ possibles, the combinadic unranking algorithm [29] is used at the transmitter. The time and space complexities of this algorithm are O(n) and O(nk), respectively [29,36,37]. However, in the proposed NSC-OFDM-IM scheme, there is no need to determine the locations of active subcarriers since the index selector starts by assuming all subcarriers as if they are active in the subgroup, and hence, it targets the deactivation of one of the active subcarriers depending on the decimal value of the incoming $p_{0}$ bit substream;
- Receiver complexity: Both waveforms, OFDM-IM and NSC-OFDM-IM, have the same LLR processing, which is applied to the frequency-domain-converted signal. In the OFDM-IM scheme, the coefficients in each LLR subgroup are first sorted in order to extract the most possible occurrence of the active subcarriers. There are different types of sorting algorithms, and each has a different computational complexity. The most commonly used sorting algorithms with their worst- and best-case complexities [38,39] are shown in Table 4. From the output of the sorting algorithm, the number of the first *k* indexes are chosen as the active carrier indexes. Then, the next step is to apply the combinadic ranking algorithm [29] to this index vector so that the corresponding integer value of the index sequence is obtained based on the lexicographic order. The complexity of this combinadic algorithm is given as $O(k^{2})$ [29,36,37]. However, in the proposed scheme, we do not need to apply the sorting and combinadic ranking algorithms at the receiver. Instead, the index detector just finds the index of the minimum value of the coefficients in each LLR subgroup. Then, by taking one less than this index number, the corresponding decimal value of the index bits is calculated. The complexity of finding the minimum value is O(n). Table 5 compares the computational complexity of the proposed NSC-OFDM-IM with several existing OFDM-IM schemes.

## 7. Simulation Results

In this section, we present the experimental results of the proposed NSC-OFDM-IM along with conventional OFDM and existing OFDM-IM. The performance of the proposed method and the conventional systems was evaluated in terms of spectral efficiency and bit error rate. The parameters in Table 6 were used for the computer simulations. The system was designed with 128-point subcarriers, which were used for the complex and index modulations, in the frequency domain.

In all waveforms, the length of the cyclic prefix was chosen as 32 to prevent ISI. As a wireless channel environment, the flat fading Rayleigh multipath channel was used in the presence of additive white Gaussian noise.

The complex data were generated by the BPSK, 4-QAM, and 8-PSK constellation mappers, and they were used to modulate the active subcarriers in all waveforms. Since the receiver has perfect channel state information (CSI), the system does not need a pilot symbol insertion in the transmitted signals for the channel estimation process. All the subcarriers are active in the conventional OFDM signal. In the proposed NSC-OFDM-IM and existing OFDM-IM schemes, 128 carrier locations are divided into *K* complex subgroups with a size of $\tilde{M}$, which is also equal to *n*. The complex subgroups were designed by using 4,8, and 16 subcarrier locations for the computer simulations. Along the frequency axis, the number of complex subgroups *K* were selected as 32,16, and 8, corresponding to the $\tilde{M}$ of 4,8, and 16 subcarriers, respectively. In the proposed method, in each complex subgroup, one carrier was set to inactive to convey the $p_{0}$ bits to the index of this null subcarrier. The total number of bits conveyed by the indices of the null subcarriers was obtained by $K(log_{2}\tilde{M})$.

**Figure 8:**
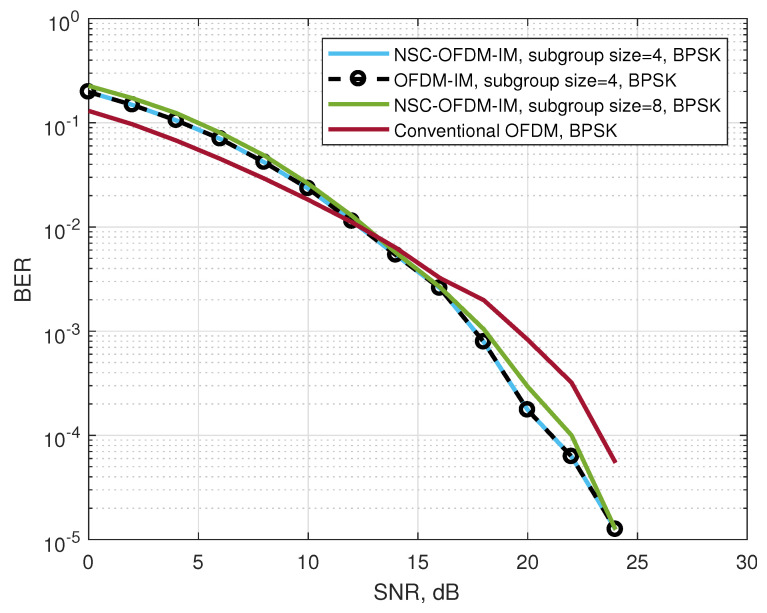
BER performance comparison between the proposed NSC-OFDM-IM and conventional OFDM-IM/OFDM, where the BPSK modulation was adopted.

Figure 8 depicts the bit error rate performance of the conventional and the proposed waveforms by using the BPSK complex modulation. The proposed NSC-OFDM-IM was tested with different sizes of complex subgroups. Each simulation result was compared with the conventional OFDM and OFDM-IM systems. The complex subgroups with the sizes of $n=\tilde{M}=4$ and $n=\tilde{M}=8$ were simulated. Along the frequency spectrum, the number of total subgroups (K) were 32 and 16 for $\tilde{M}=4$ and $\tilde{M}=8$, respectively. In the low SNR values, the proposed system struggled to achieve the null carrier indices due to the significant amount of noise, which caused the signal to be distorted, at the position of the null subcarriers in the frequency domain. However, the performance of the system was significantly improved by the increasing SNR values while maintaining the same BER as OFDM-IM.

**Figure 9:**
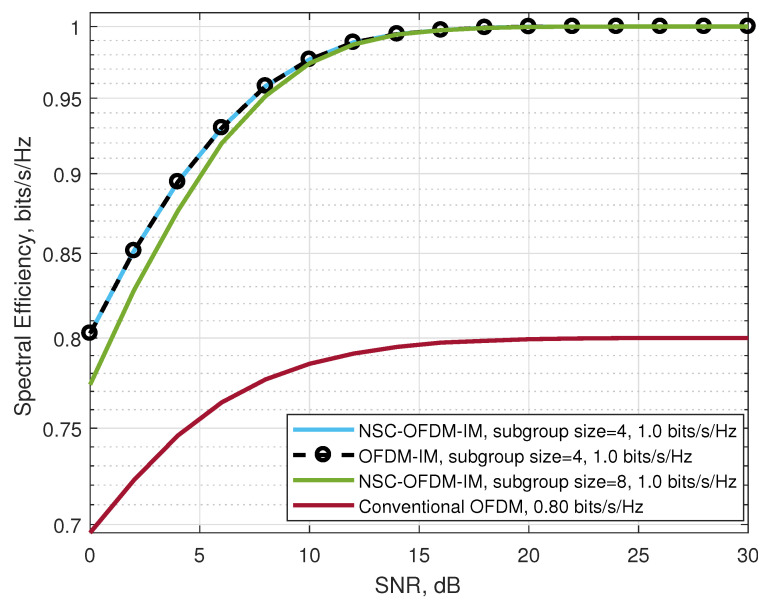
Comparison of the proposed NSC-OFDM-IM with conventional OFDM/OFDM-IM in terms of spectral efficiency, where the BPSK modulation was adopted.

In Figure 9, the spectral efficiencies of the waveforms in the BPSK modulation are illustrated. Theoretically, in terms of the spectral efficiency, as shown in Table 3, the proposed scheme outperformed the conventional OFDM while having the same spectral efficiency as the existing OFDM-IM. Experimentally, we showed that the proposed NSC-OFDM-IM scheme had an improved performance over the increasing SNR values as compared to the conventional OFDM scheme. From Table 3, we also observed that the NSC-OFDM-IM scheme had the same spectral efficiency in different complex subgroup configurations, which consisted of three and four subcarrier locations. However, according to the simulation results of each NSC-OFDM-IM configuration, the system designed with the complex subgroups having four subcarrier locations reached the maximum spectral efficiency earlier than the other configuration consisting of eight subcarrier locations in the complex subgroups.

**Figure 10:**
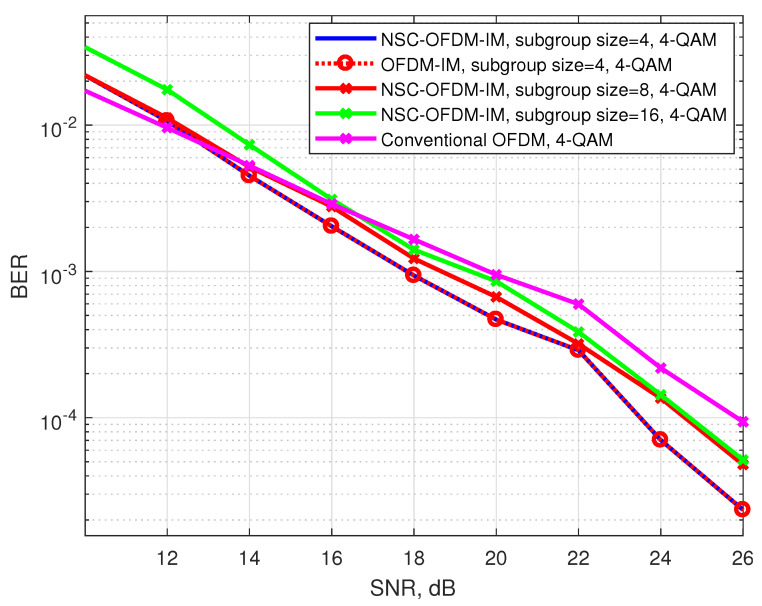
BER performance comparison between the proposed NSC-OFDM-IM and conventional OFDM-IM/OFDM, where 4-QAM modulation was adopted.

Figure 10 shows the comparison of the bit error rates by using the 4-QAM complex modulation. The proposed system was tested by configuring the complex subgroups with 4, 8, and 16 subcarriers.

Theoretically, in the design of the complex subgroups with four subcarriers, the proposed system had the same spectral efficiency as the classical OFDM system. However, according to the simulation results, the proposed waveform exhibited better BER performance than the OFDM system. Most probably, the reason for this behavior was that the proposed waveform successfully captured the null carrier locations; however, conventional OFDM failed to obtain the complex data, which coincided at the same location as the NSC-OFDM-IM null carriers along the frequency axis. It was also observed that the proposed scheme achieved the same error performance as the OFDM-IM scheme.

**Figure 11:**
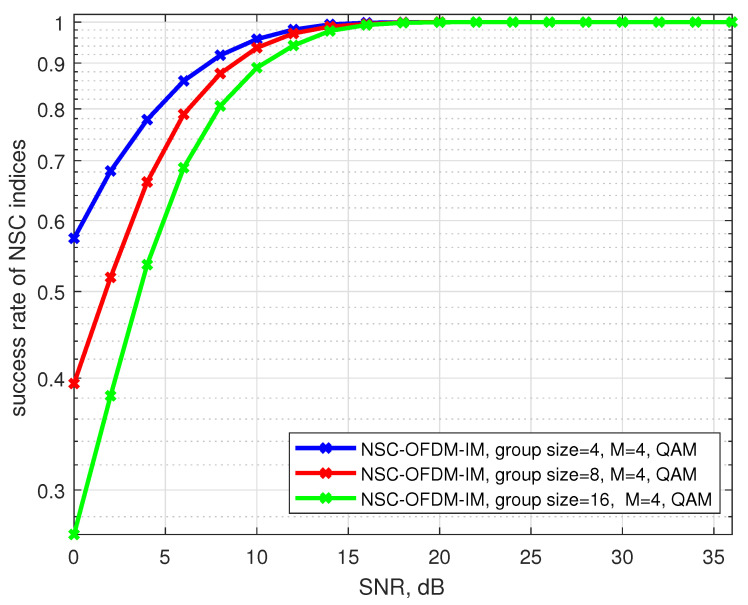
Succession rate of the null carrier indices for the proposed NSC-OFDM-IM system.

The succession rate of the null subcarrier indices was also affected by the low SNR values and the size of the subgroups. Figure 11 shows the succession rate of capturing the correct null carrier positions in the complex subgroups. The system configured by using four subcarrier locations in each subgroup had better succession performance than the other subgroup configurations consisting of eight and sixteen subcarrier locations, in lower SNR values. However, with the increasing SNR regimes, all the inactive carrier positions along the frequency spectrum were successfully captured in all 4-QAM modulated subgroup configurations.

**Figure 12:**
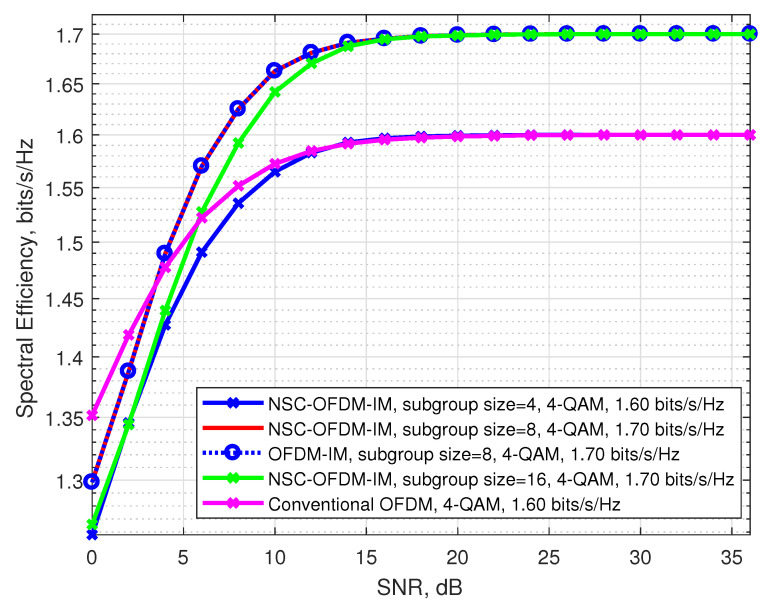
Comparison of the proposed NSC-OFDM-IM with conventional OFDM/OFDM-IM in terms of spectral efficiency, where 4-QAM modulation was adopted.

The spectral efficiencies of the proposed and the conventional waveforms by using the 4-QAM modulation are illustrated in Figure 12. In these simulations, we see that the spectral efficiency of the proposed scheme was also experimentally achieved by comparing to conventional OFDM and OFDM-IM systems.

We also observed that the waveforms designed with the structure of complex subgroups that were configured by using eight and sixteen subcarriers theoretically had the same spectral efficiency as shown in Table 3. However, the performance of the waveforms was affected by the complex subgroup size, at low SNR values: the waveform configured with the complex subgroups that consisted of eight carrier locations exhibited better spectral efficiency than the other waveform configured with the subgroups that had sixteen carrier locations.

**Figure 13:**
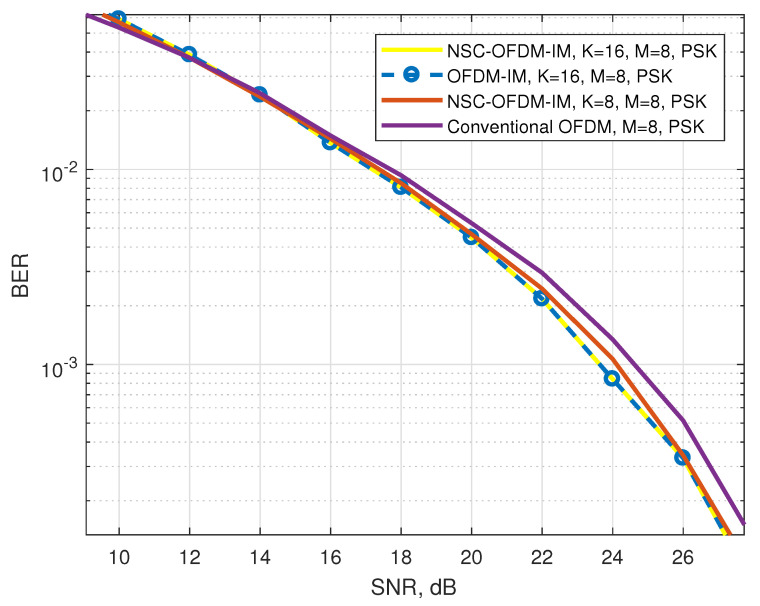
BER performance comparison between the proposed NSC-OFDM-IM and conventional OFDM-IM/OFDM, where 8-PSK modulation was adopted.

**Figure 14:**
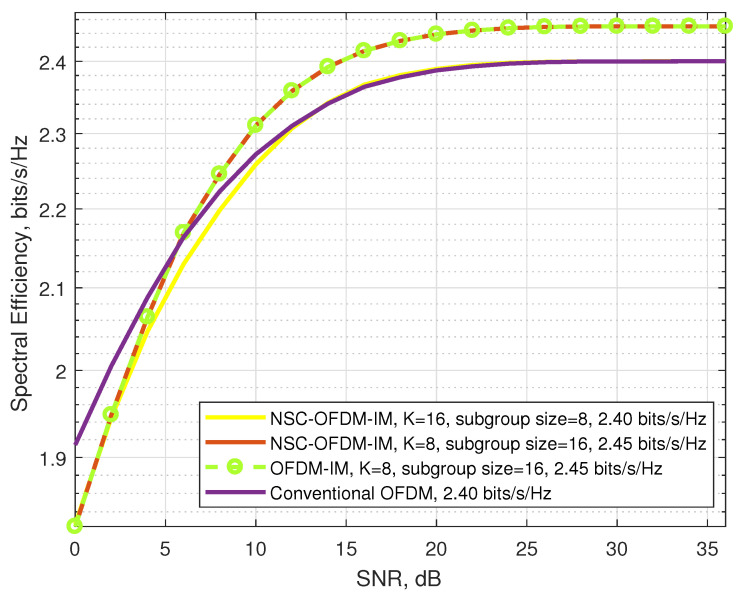
Comparison of the proposed NSC-OFDM-IM with conventional OFDM/OFDM-IM in terms of spectral efficiency, where 8-PSK modulation was adopted.

In Figure 13, the conventional and the proposed NSC-OFDM-IM waveforms were simulated by using the 8-PSK complex modulation. The complex subgroups were configured with eight and sixteen subcarrier locations for index-modulated waveforms. Looking into the bit error rate performance, the performance of the index-modulated scheme was affected by the complex modulation order, and it expressed slightly better bit error rate performance than the conventional OFDM. Moreover, in terms of spectral efficiency, the system had better performance in the increasing SNR values, as shown in Figure 14.

In order to better illustrate the motivation for using the log-likelihood ratio mechanism for the null subcarrier index detection, we also simulated our proposed NSC-OFDM-IM waveform without the LLR design at the receiver, as illustrated in Figure 5. Instead, we designed the index detection mechanism just after the frequency domain equalization. The equalized spectrum was first divided into a number of K complex subgroups, and then, by taking the absolute values of the complex coefficients in each complex subgroup, we extracted the index of the minimum absolute value among them. The index number was assumed to belong to the null subcarrier location within the complex subgroup.

**Figure 15:**
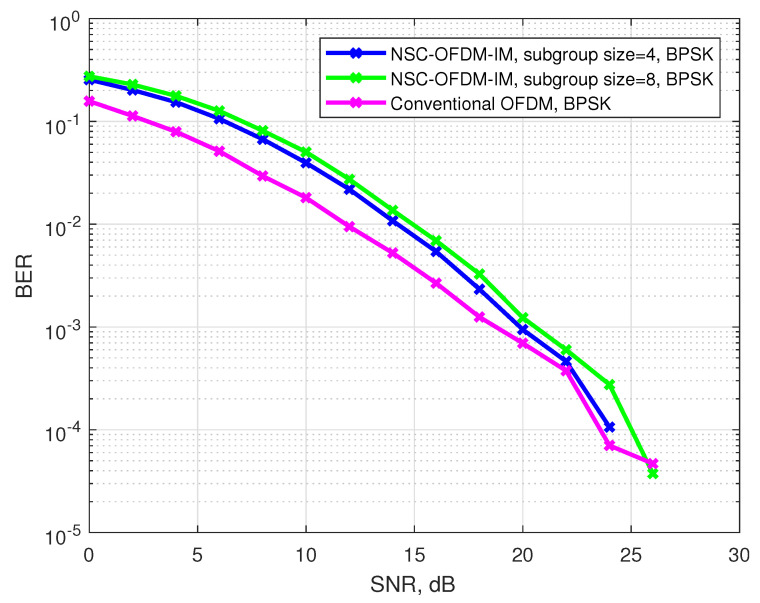
Performance of the proposed NSC-OFDM-IM without applying the LLR detection.

Figure 15 depicts the BER performances of classical OFDM and the proposed NSC-OFDM-IM (without the LLR detector) waveforms using the BPSK complex modulation and the complex subgroup designs with four and eight subcarriers.

In the simulations, we observed that the OFDM signal expressed better performance than the proposed scheme. This was due to the detection mechanism in NSC-OFDM-IM, which does not take into account the channel noise while detecting the null carrier index, hence causing a high number of detection failures at the null subcarrier positions. By comparing it to conventional OFDM, while both waveforms aim to obtain the complex modulated data on the active subcarriers, the index modulated waveform was also struggling to detect the null carrier indices in a noisy environment in this case. In summary, the performance of index modulation scheme was highly affected by the misdetection of null carrier indices.

## 8. Discussion

Computational complexity is one of the open issues in the existing OFDM-IM systems. In this paper, we proposed a new method for mapping and detecting the index bits with the reduced computational complexity algorithms. With the aim of overcoming the problems mentioned in the Introduction, the proposed method tackles the complexity problem by eliminating the need for using either a look-up table or a combinatorial algorithm for the index mapper and detector. Different from the existing studies [7,8,9,10,11,12,13,14,15,16,17] on the OFDM-IM scheme, in the frequency domain subgroup, this novel transmission technique incorporates the index of a null subcarrier rather than the indexes of active subcarriers. The additional data bits (the index bits) are conveyed by the index of the null subcarrier in the subgroup.

We showed that the computational complexity of the proposed method was significantly reduced as compared with the known OFDM-IM scheme, as shown in Table 5. The proposed NSC-OFDM-IM also maintains the spectral efficiency of the existing OFDM-IM scheme, as depicted in Table 3. Moreover, this new method does not suffer from any performance degradation and expresses the same BER performance, as illustrated in Figure 8, Figure 10, and Figure 13, but it differs from the existing studies [11,14] in terms of the need to use the combinadic algorithms and the way of conveying the index bits through the indices of the subcarrier locations. Furthermore, while these existing studies express the computational complexity of the index selection/detection algorithms (the combinatorial algorithms), the proposed method in our study does not require using the combinatorial method.

At the transmitter side, the complex subgroup in the frequency domain was designed in such a way that the proposed method first assumes all subcarriers as if they are all in the active state in the frequency domain, and then, based on the incoming index bits, it starts by deactivating one of the active subcarriers within the subgroup. In other words, we were interested in finding the location of the subcarrier that will be left empty in the frequency domain, which means there will be no carrier generated at that frequency point. The remaining active subcarriers were used for the complex data modulation, as in the conventional OFDM-IM systems. In contrast to the existing OFDM-IM methods, the proposed method is not concerned with the order of the active subcarrier indices in the complex subgroup, since these indices are not taken into account for data transmission.

At the receiver side, the proposed NSC-OFDM-IM has the same LLR processing as the existing OFDM-IM, but the detection of the subcarrier index is performed in a different way in comparison to the work in [13,17]. In more detail, in the traditional OFDM-IM scheme, the LLR algorithm is used to find the arrangement of the indices of the active subcarriers in the complex subgroup. However, in the proposed NSC-OFDM-IM scheme, the index detector algorithm is only interested in finding the null subcarrier location in the subgroup since the extra information bits are mapped to the inactive subcarrier index. In other words, the minimum value of the LLR coefficients in each subgroup indicates that the probability of occurrence of an active subcarrier at that location (the index of minimum value) is extremely small, and hence, it means the occurrence of a null subcarrier.

## 9. Conclusions

In this study, a novel index modulation method called NSC-OFDM-IM was introduced. The modulation was carried out for a $\tilde{M}$-size subgroup, which had a number of $\tilde{M}-1$ active subcarriers. Unlike in the existing OFDM-IM schemes, where the index selection is performed for the purpose of determining the active subcarriers in the subgroup, in the proposed NSC-OFDM-IM scheme, the null subcarrier location is selected to convey the transmitted $p_{0}$ bits to the null subcarrier index. It was shown that the combinadics algorithms for the index selection and index detection were no longer required since the proposed method utilizes the null subcarriers rather than active subcarriers. Thus, the NSC-OFDM-IM scheme has a significantly reduced complexity as compared to the existing OFDM-IM system. Simulations and analyses were carried out in terms of spectral efficiency and bit error rate performances, and the results were compared with the OFDM and OFDM-IM systems. The proposed scheme had better spectral efficiency and bit error rate performances than the conventional OFDM system, without causing any performance degradation to the existing OFDM-IM scheme.

The structure of the complex subgroups, whose sizes were chosen as a power of two, allows an easy and flexible design of the scheme at the transmitter and receiver. The proposed method and presented results motivate future works on designing a low-complexity and flexible index selection and index detection algorithms for the transmitter and receiver systems. This approach is a novel way of representing data transmission using the null subcarrier indices, and it outperformed the existing OFDM-IM system in terms of reducing the computational complexity without the need for a look-up table or combinatorial algorithms for index mapping/demapping operations. In our future studies, we will combine the proposed method with a reduced-complexity LLR detection mechanism so that the complexity can be further decreased to improve the performance of the detection at the receiver.

## Figures and Tables

**Table 1:** The proposed scheme index modulator.

Incoming Bits ($p_{0}$)	Bits Decimal Value (Decvalue)	Null Subcarrier Index (Location in Complex Subgroup)
{00}	0	decvalue+1=1
{01}	1	decvalue+1=2
{10}	2	decvalue+1=3
{11}	3	decvalue+1=4

**Table 2:** The proposed scheme index demodulator ($\tilde{M}$ = 4).

Null Carrier Index	Corresponding	Corresponding
(Extracted from LLR Subgroup ($\tilde{i}$))	Decimal Value ($\tilde{z}$)	Binary Sequence (${\tilde{p}}_{0}$)
1	$\tilde{i}-1=0$	${\tilde{p}}_{0}=\left\{00\right\}$
2	$\tilde{i}-1=1$	${\tilde{p}}_{0}=\left\{01\right\}$
3	$\tilde{i}-1=2$	${\tilde{p}}_{0}=\left\{10\right\}$
4	$\tilde{i}-1=3$	${\tilde{p}}_{0}=\left\{11\right\}$

**Table 3:** Spectral efficiencies of OFDM, OFDM-IM, and NSC-OFDM-IM ($N_{FFT}=128$, $N_{cp}=32$, $n=\tilde{M}$, and $k=\tilde{M}-1$).

Waveforms	Complex Group Size ($\tilde{M}$)	BPSK		4-QAM		8-PSK	
		Spectral Efficiency	Extra Bits	Spectral Efficiency	Extra Bits	Spectral Efficiency	Extra Bits
Classical OFDM	-	0.80	-	1.60	-	2.40	-
NSC-OFDM-IM/OFDM-IM	2	0.80	0	1.2	−1	1.60	−2
NSC-OFDM-IM/OFDM-IM	4	1	+1	1.60	0	2.20	−1
NSC-OFDM-IM/OFDM-IM	8	1	+2	1.70	+1	2.40	0
NSC-OFDM-IM/OFDM-IM	16	0.95	+3	1.70	+2	2.45	+1
NSC-OFDM-IM/OFDM-IM	32	0.90	+4	1.68	+3	2.45	+2

**Table 4:** Computational complexities of commonly used sorting algorithms.

Sorting Algorithms	Time Complexity			Space Complexity
	Best Case	Average Case	Worst Case	
Quick	O(nlogn)	O(nlogn)	$O(n^{2})$	O(nlogn)
Merge	O(nlogn)	O(nlogn)	O(nlogn)	O(n)
Heap	O(nlogn)	O(nlogn)	O(nlogn)	O(1)
Insertion	O(n)	$O(n^{2})$	$O(n^{2})$	O(1)
Bubble	O(n)	$O(n^{2})$	$O(n^{2})$	O(1)

**Table 5:** Complexity comparisons between NSC-OFDM-IM and OFDM-IM schemes.

Operations	OFDM-IM		NSC-OFDM-IM	
	Time Complexity	Space Complexity	Time Complexity	Space Complexity
Combinadic Unranking (Tx)	O(n)	O(nk)	−	−
Combinadic Ranking (Rx)	O(n)	$O(k^{2})$	−	−
Sort LLR values (Quick-Rx)	O(nlogn)	O(nlogn)	−	−
Find min value index (Equation (21))	−	−	O(n)	O(1)

**Table 6:** The simulation parameters.

Parameters	Parameter Value
FFT Size	128
Number of used subcarrier locations	128
Cyclic Prefix Length	32
Channel type	Rayleigh fading + AWGN
Number of channel taps	20
Complex subgroup sizes	4, 8, 16
Complex modulations	BPSK, 4-QAM, 8-PSK

## Data Availability

Not applicable.
